# Understanding Effects of Ultrasonic Vibration on Microstructure Evolution in Hot Forming Process via Cellular Automata Method

**DOI:** 10.3390/ma15207359

**Published:** 2022-10-20

**Authors:** Yutong Zhang, Weihua Zhou, Jinyuan Tang, Yuhui He

**Affiliations:** State Key Laboratory of High Performance Complex Manufacturing, Central South University, Changsha 410023, China

**Keywords:** ultrasonic-assisted thermal forming, ultrasonic softening effect, stress reduction amplitude, evolution of microstructure

## Abstract

Compared with traditional forming technology, ultrasonic vibration-assisted plastic forming technology can improve the forming conditions and obtain better surface quality of the workpiece. However, the mechanism and theory of ultrasonic action have not formed a unified understanding. In this paper, ultrasonic-assisted thermal forming technology is taken as the research object. Through experimental research combined with cellular automata methods, based on the dislocation density model, nucleation and growth model, and dynamic recrystallization growth rule, a theoretical model for microstructure simulation of the ultrasonic-assisted thermal forming process was established. By introducing the ultrasonic energy field into the thermal forming process and correcting thermal activation energy and dynamic recovery coefficient, the reasons for flow stress reduction of 9310 steel and the influence of temperature, strain rate, and vibration amplitude on recrystallization were analyzed from the microscopic scale. The results show that the introduction of ultrasonic vibration reduces the dislocation activation energy, promotes dynamic recrystallization behavior, and finally leads to the reduction of flow stress. With an increase in vibration amplitude, the average grain size decreases faster, the recrystallization volume fraction increases faster, the stress decreases larger, and the ultrasonic softening phenomenon becomes more obvious. Decreasing the strain rate will promote the occurrence of dynamic recrystallization, the volume fraction and average grain size of dynamic recrystallization will increase, and the true stress will decrease.

## 1. Introduction

Compared with the traditional forming process, ultrasonic-assisted thermal forming can reduce the forming load and flow stress of the material, refine the grain, and obtain a workpiece with good forming quality [1]. Although ultrasonic vibration-assisted plastic-forming technology has been widely used in processing technology, there is no unified understanding of the internal mechanism of ultrasonic vibration [2,3].

After applying ultrasonic vibration, the material softens, and the flow stress decreases, which is known as the ultrasonic softening effect [4,5]. Wen [6] studied the plastic behavior of AZ31 magnesium alloy during the room temperature tensile process under ultrasonic vibration and revealed the volume effect of vibration plastic deformation. The ultrasonic softening effect reduces flow resistance and improves plasticity. When a lower amplitude or vibration energy is applied to the tensile specimen, the softening effect is dominant, resulting in a decrease in the deformation resistance of AZ31 with an increase in formability. Zhou [7] conducted an ultrasonic-assisted compression (UAC) experiment to study the influence of ultrasonic softening on the plasticity of aluminum and titanium by changing vibration amplitudes and frequencies. The experimental results show that ultrasonic vibration can reduce the flow stress of aluminum and titanium in the UAC process. In the range of 20–40 kHz, increasing the vibration amplitude can enhance the ultrasonic softening effect, and increasing the vibration frequency will reduce the ultrasonic softening effect.

The softening effect of ultrasonic is manifested as the decrease in flow stress at the macro level, and it affects the plastic-forming mechanism and microstructure of the material at the micro level [8,9]. Most of the studies on microstructure evolution under the action of ultrasonic vibration use metallographic microscopy or EBSD technology to observe the evolution of microscopic tissues during the application of ultrasonic vibration after ultrasonic vibration physics experiments. Hung [10,11] performed an ultrasonic vibration upsetting experiment and metallographic analysis of an A6061-T6 aluminum alloy. The experimental results showed that the surface grain of the sample is refined when ultrasonic vibration is superimposed on the sample. Zhao [12] studied the influence of ultrasonic vibration applied in three directions on the mechanical behavior and microstructure of steel plates in the process of tensile deformation. The radial vibration results in a significant increase in low-angle grain boundaries and the formation of subgrain boundaries in grains, and the axial and normal vibration results in a decrease in the proportion of low-angle grain boundaries and a decrease in dislocation density.

To study the reason for grain refinement caused by ultrasonic vibration, a large number of scholars have analyzed and explained it through experiments. Currently, a generally accepted explanation is that ultrasonic vibration increases the nucleation rate and promotes dislocation movement. Hu [4] used pure copper as the research object and conducted ultrasonic-assisted compression tests at different amplitudes and found that the stress reduction caused by ultrasonic softening increased with the increase of flow strain or ultrasonic amplitude. EBSD observation shows that when ultrasonic softening occurs, low-angle grain boundaries are randomly distributed in grains, while when no ultrasonic vibration is applied, they show a stacking distribution, which means that acoustic softening improves the movement of small-angle grain boundaries or dislocations, thus reducing flow stress. In addition, at small deformation strains, elongated grains become equiaxed, and the dislocation density is significantly reduced, which may be the result of increased dislocation annihilation due to ultrasound-induced dynamic recovery. However, with the increase in deformation strain, ultrasonic hardening gradually becomes significant, resulting in a greatly reduced effect of acoustic softening on the reduction of dislocation density. Zhou [7] conducted an ultrasonic-assisted compression experiment and an EBSD experiment on aluminum and titanium samples along the compression and transverse directions. The EBSD results show that the upper plate area of the aluminum sample is more sensitive to the influence of ultrasonic vibration than the center of the sample. Transient ultrasonic vibration increases the subgrains in the aluminum sample, and the induced grain boundaries with small angles are often parallel to the vibration direction. For titanium samples, ultrasonic vibration mainly affects the central area of the sample, and transient ultrasonic vibration also increases the generation of subgrains and twinning. This indicates that the refinement of grains is achieved through the proliferation of subgrains and that subgrains can be used as an indicator of dislocation evolution. Liu [13] conducted an ultrasonic vibration upsetting experiment and found that the dislocation movement in the area of severe deformation was intense and that the dislocation was intertwined, resulting in a dislocation wall. Finally, these dislocation walls become grain boundaries with small or large angles, and then the elongated grains are refined into small grains and subgrains. This indicates that ultrasonic vibration promotes dislocation movement. Wang [14,15] studied the evolution characteristics of grain size, orientation angle, and texture under different amplitudes, and found that there was a positive correlation between the number of subgrains, low-angle grain boundaries, and amplitude. EDS, XRD, and EBSD analysis revealed the evolution mechanism of alloy microhardness, and it was found that the microhardness of the Ti-45Nb sample increased first and then decreased with an increase in amplitude.

At present, most studies on microstructure evolution under ultrasonic vibration are based on the analysis of experimental phenomena, and the reasons for grain refinement and rheological stress reduction under ultrasonic vibration have not been unified. Cellular automata (CA) [16] is a grid dynamic model with discrete time, space and state, local spatial interaction, and temporal causality. It can simulate the spatiotemporal evolution of complex systems and can be used to simulate and analyze the process of ultrasonic-assisted thermal forming. Steel 9310 has high hardenability, high hardness, and high fatigue strength. It is widely used for aero engine gear and steam turbine gear. In the rough machining process of gear, high-temperature forging is often adopted, so 9310 steel is selected in this paper as a material for high-temperature deformations. This paper is based on a thermal simulation experiment and an ultrasonic vibration-assisted tensile experiment and studies the microstructure evolution of ultrasonic-assisted thermal forming. The cellular automata simulation model of ultrasonic-assisted thermal forming is established, and the reasons for grain refinement and flow stress reduction are further discussed at the microscopic scale.

## 2. CA Model

### 2.1. Grain Growth

In the process of hot compression, when the holding temperature is above the austenitizing temperature, the grains undergo a grain growth process, so the CA model of grain growth is first established. In this paper, the cell size ($L_{ca}$) is 2 μm, and the simulated space size is 200 × 200; the actual simulated area is 0.4 cm × 0.4 cm. The neighbor type is a six-neighbor. The state variable of the cellular is grain orientation. The number of grain orientations ranged from 1 to 180. In the initial state, all cells in the cell space are given a random integer from 1 to 180 as orientation, which is taken as the initial state of the cellular space. There are three main rules for the transformation of the cellular state:

(1) Thermodynamic energy mechanism. The normal grain growth process is essentially a process of thermal activation. Grain boundary migration can occur only when grain boundary atoms overcome certain energy barriers. When the thermal energy of the cell at the grain boundary is greater than the activation energy of thermal diffusion, its state can change. According to statistical thermodynamic theory, the probability that the thermal energy of the cell at the grain boundary is greater than the activation energy of thermal diffusion can be calculated as follows [17]:

(1) $$P_{1}=A\cdot \exp(-\frac{Q_{b}}{RT})$$

where *A* is the material parameter, which can be calculated from $P_{1}=1$ when the temperature reaches the melting point; *T* is the temperature (K); *Q_b_* is the thermal diffusion activation energy; *R* is the molar gas constant; and the value is 8.31 J/(mol·K).

(2) Curvature drive mechanism. According to the orientation relationship and grain boundary type of neighbor cells, the current judging cell is regarded as the central cell. If four or more neighbor cells have the same state “A”, while the central cell state is different from them, the central cell state will change into “A” state at the next time step.

If there are three neighboring cells in the same state “B”, while the central cell is different, then the transition probability $P_{2}=0.4$ will be used to judge. If it is satisfied, the central cell state will change to a “B” state; otherwise, the state is unchanged.

If the currently judged cellular does not satisfy the curvature drive mechanism, the next criterion will be used to judge.

(3) The principle of least energy. According to the principle of energy dissipation, normal grain growth is a process in which the grain boundary expands outward and the grain boundary energy decreases. Since hexagonal cellular can simulate isotropy well, the calculated grain boundary energy also has isotropy, and the Hamiltonian function is used to represent the grain boundary energy [18]:

(2) $$E_{i}=J\overset{N}{\underset{k}{\sum }}\left(1-\delta_{C_{i}C_{k}}\right)$$

where *J* is the grain boundary energy coefficient, the value is 1; *N* is the number of neighbors of the cellular, the value is 6; *k* is the neighbor of the current judged cell; *δ* is the Kronecher symbol; $C_{i}$ is the current orientation of the cell; $C_{k}$ is the orientation value of the neighbor cell of the current judgment cell.

Judging the 6 neighboring cells in random order, the change of grain boundary energy can be calculated as follows:

(3) $$\Delta E_{i\to j}=E_{j}-E_{i}=J\overset{N}{\underset{k}{\sum }}\left(1-\delta_{C_{j}C_{k}}\right)-J\overset{N}{\underset{k}{\sum }}\left(1-\delta_{C_{i}C_{k}}\right)$$

Whether the state of the center cell changes to the state of the neighbor cell can be determined by judging whether the change in the grain boundary energy of the system is less than 0. If ΔE<0, the state of the center cell changes to the neighbor cell; If ΔE>0, the central cell state does not change.

In one time step, the grain boundary cells in the cell space are judged to determine whether the state changes according to the above order. 

When all grain boundary cells in the cellular space are judged, update the state variables of each cell under this time step. Then, determine the grain boundary cells according to the orientation value, plot the microstructure image, and calculate the average grain size. Stop when the average grain size meets the set conditions; otherwise, the time step is added to one for the next round of judgment.

### 2.2. Dynamic Recrystallization with Ultrasound

The results obtained by the normal grain growth model are used as the initial cellular space state for the dynamic recrystallization CA simulation. In the simulated dynamic recrystallization process, the state variables of the cellular include grain orientation, dislocation density, number of recrystallizations, state variables reflecting the new and old frames, and state variables regarding whether the cell is located at the boundary. 

#### 2.2.1. Dislocation Density Model

To describe the change in dislocation density inside the material, the dislocation density evolution model proposed by KCOKS and MECKING is adopted, as shown in Equation (4) [19]:

(4) $$\frac{d\rho }{d\varepsilon }=k_{1}\sqrt{\rho }-k_{2}\rho$$

where ρ is dislocation density; *ε* is strain; $k_{1}$ is the dislocation increment coefficient, $k_{2}$ is the dislocation extinction coefficient. The hardening index *C* can be obtained from the experimental data by Formula (5), and $k_{1}$ and $k_{2}$ can be obtained by combining Formulas (6) and (7):

(5) $$\frac{d\sigma }{d\varepsilon }=C\left(1-\frac{\sigma }{\sigma_{s}}\right)$$

(6) $$C=0.5\mu bk_{1}/2$$

(7) $$\sigma_{s}=0.5\mu b\left(k_{1}/k_{2}\right)$$

The flow stress can be calculated using Equation (8):

(8) $$\sigma =\alpha \mu b\sqrt{\bar{\rho }}$$

where *σ* is the flow stress; *α* is the material constant, the value is 0.5; *μ* is the shear modulus; $\bar{\rho }$ is the average dislocation density. The initial dislocation density $\rho_{0}$ in the process of thermal simulation can be obtained by substituting the initial yield stress $\sigma_{0}$ into Equation (8).

The time step (Δt) can be calculated as follows [20]:

(9) $$\Delta t=\frac{{2k}_{1}{}^{2}Lca}{\mu b^{2}Mk_{2}{}^{2}}$$

At each time step, the strain and dislocation density is calculated according to the following equation:

(10) $$\varepsilon^{t+\Delta t}=\varepsilon^{t}+\dot{\varepsilon }\Delta t$$

(11) $$\rho^{t+\Delta t}=\rho^{t}+\Delta \rho$$

where $\dot{\varepsilon }$ is the strain rate.

With the increase in strain, the dislocation density increases, and when it reaches the critical dislocation density, dynamic recrystallization grain nucleation occurs at the grain boundary. The critical dislocation density ($\rho_{cr}$) can be calculated as follows [21]:

(12) $$\rho_{cr}={\left(\frac{20\gamma_{i}\dot{\varepsilon }}{3blM\tau^{2}}\right)}^{1/3}$$

where $\gamma_{i}$ is the grain boundary energy, which can be obtained by Equation (13); *M* is the grain boundary migration energy, which can be obtained by Equation (15); τ is the dislocation line energy, which can be calculated by Equation (16); and *l* is the line dislocation energy, which can be obtained by Equation (17).

(13) $$\gamma_{i}=\{\begin{array}{l} \gamma_{m},\theta_{i}\geq 15{}^{\circ}\\ \gamma_{m}\frac{\theta_{i}}{\theta_{m}}\left(1-\ln(\frac{\theta_{i}}{\theta_{m}}\right)),\theta_{i}\leq 15{}^{\circ} \end{array}$$

(14) $$\gamma_{m}=\frac{\mu b\theta_{m}}{4\pi \left(1-v\right)}$$

where $\gamma_{m}$ is the grain boundary energy of large angle grains; $\theta_{m}$ is the orientation angle of large Angle grains; $\theta_{i}$ is the orientation difference; v is Poisson’s ratio.

(15) $$M=\frac{D_{ob}b}{k_{2}T}e^{-\frac{Q_{b}}{RT}}$$

(16) $$\tau =0.5\mu b^{2}$$

(17) $$l=\frac{k_{1}\mu b}{\sigma }$$

where R is the gas constant; $Q_{b}$ is the diffusion activation energy of grain boundaries; b is the Berkovian vector; μ is the shear modulus; $D_{ob}$ is the self-diffusion coefficient.

To study the reasons for grain refinement caused by ultrasonic vibration, a large number of scholars have made explanations through experimental phenomena and metallographic analysis. The widely accepted explanation is that ultrasonic vibration improves the nucleation rate and promotes dislocation movement. Ultrasonic vibration acts inside the material, and the defect is easy to absorb ultrasonic energy, so the activation energy required for dislocation movement is reduced after ultrasonic vibration is applied. According to this principle, the influence of ultrasonic was introduced into the thermal activation model, and the energy reduced by ultrasonic was subtracted from the thermal activation energy to obtain the thermal activation model under ultrasonic vibration. Since ultrasonic energy cannot be completely absorbed by the material defect, correction factor C is introduced. The activation energy under ultrasonic vibration can be obtained from the following equation:

(18) $$Q_{b}^{\prime }=Q_{b}-C_{2}E_{u}$$

where $Q_{b}^{\prime }$ is the activation energy after applying ultrasonic vibration; $C_{2}$ is the correction coefficient, which can be obtained by fitting the results of the ultrasonic-assisted tensile experiment, and is 0.4 in this paper; $E_{u}$ is ultrasonic energy, which can be calculated by Formula (19):

(19) $$E_{u}=A^{2}\omega^{2}\;\rho_{1}$$

where *A* is the ultrasonic vibration amplitude; ω is the vibration circle frequency, and ω=2πf, where f represents the ultrasonic vibration frequency, $\rho_{1}$ is the density.

After adjusting the grain boundary migration energy, the expression of the grain boundary migration energy under ultrasonic vibration is obtained as:

(20) $$M_{u}=\frac{D_{ob}b}{k_{2}T}e^{-\frac{Q_{b}^{\prime }}{RT}}$$

According to the relevant experimental results of ultrasonic-assisted stretching, the true stress–strain curve after the application of ultrasound is not completely parallel to that without the application of ultrasound, which indicates that the internal structure or microstructure of the metal material changes after the application of ultrasonic vibration. Therefore, the parameters in the dislocation density model were adjusted to obtain the dislocation density model under applied ultrasonic vibration.

Modify the parameter $k_{2}$ of dynamic recovery part in the dislocation density model in Equation (4), which is the parameter $k_{2u}$ of the dynamic recovery part under ultrasonic vibration, the calculation formula is:

(21) $$k_{2u}=k_{2}\zeta A^{\eta }$$

The ζ and η can be obtained by experimental fitting. 

When the strain increases to a certain value, the dislocation density of the material becomes stable, and the dislocation density becomes saturated. The saturation dislocation density $\left(\rho_{s}\right)$ can be obtained from Equation (22).

(22) $$\rho_{s}={\left(\frac{k_{1}}{k_{2}}\right)}^{2}$$

#### 2.2.2. Dynamic Recrystallization Nucleation and Growth Model

The nucleation model associated with dislocation density was used for the simulation. The nucleation model connects the nucleation rate with dislocation density, as shown in Equations (23) and (24). Combining with the dislocation density model, it can be seen that dislocation density is related to strain, so the nucleation rate changes with strain when the temperature and strain rate are constant.

(23) $$P_{N}=\frac{\dot{N_{l}}\Delta t}{N_{CA}}=\dot{N_{l}}\cdot L_{CA}\cdot \Delta t$$

(24) $$\dot{N_{l}}=\frac{\sqrt{\rho }-\sqrt{\rho_{\mathrm{cr}}}}{\sqrt{\rho_{s}}-\sqrt{\rho_{cr}}}$$

where $P_{N}$ is the nucleation probability; $\dot{N_{l}}$ is the grain boundary nucleation rate, which represents the number of new grains formed per unit length of grain boundaries per unit time when the dislocation density exceeds the critical value.

Dynamic recrystallization can occur many times, and the recrystallization times of newly generated recrystallized grains are increased by one. Recrystallized grains with large recrystallization times will grow into non-recrystallization grains and low-order recrystallized grains.

At each time step, non-recrystallization cells or low-order recrystallized grains located at the grain boundary are taken as the target cells, and the growth distance (*L)* of the recrystallized grains to the target cells is calculated as follows:

(25) L=Vt

where *V* is the growth rate, and the calculation formula is:

(26) $$V=M_{u}P$$

where *P* is the driving force and can be calculated as follows [22]:

(27) $$P=\frac{\mu b^{2}\left(\rho_{m}-\rho_{rex}\right)}{2}-\frac{2\gamma_{i}}{r_{i}}$$

where $\rho_{m}$ is the dislocation density of adjacent cells; $\rho_{rex}$ is the dislocation density of recrystallized grains respectively; $r_{i}$ is the radius of the recrystallization grain.

If the maximum growth distance ($L_{max}$) in a neighbor cell is larger than a cell size *L_CA_*, the target cell will be transformed into the recrystallized cell with a probability of $P_{3}=a/6$, where a is the number of cells with the same orientation number among the six neighboring cells of the cell. When the transition is successful, the state of the target cell will be consistent with that of the recrystallized cell, and its dislocation density, recrystallization number, and orientation number will be the same as that of the recrystallized cell. 

### 2.3. Program Flow Chart

**Figure 1:**
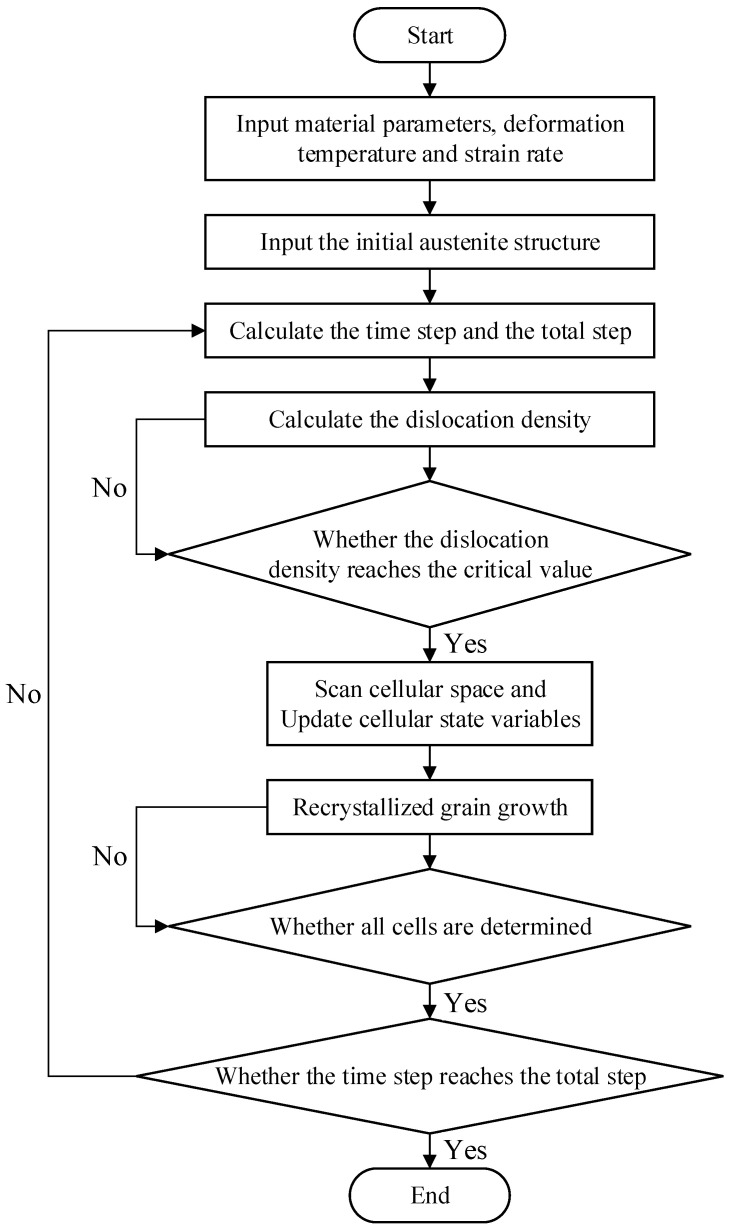
Program flow chart of dynamic recrystallization.

The simulation process of dynamic recrystallization is shown in Figure 1. 

## 3. Experiment

### 3.1. Hot Compression Experiment

**Figure 2:**
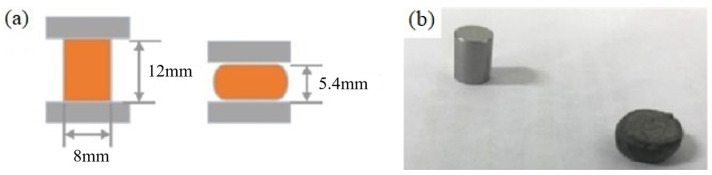
Experimental Equipment: (**a**) Schematic diagram of hot compression; (**b**) Samples before and after compression.

A Gleeble 3180 thermal simulation testing machine was used to conduct a thermal simulation test to obtain the true stress–strain curve. Gleeble 3180 is produced by Dynamic System Inc. in the US. A schematic diagram of hot compression is shown in Figure 2a. Experimental specimens before and after hot compression are shown in Figure 2b.

The thermal simulation sample is a cylinder with a diameter of 8 mm and a height of 12 mm. The test material was 9310 steel, and its chemical composition is shown in Table 1.

The first step of the thermal simulation experiment is to heat the 9310 steel cylindrical sample to the deformation temperature at a rate of 5 °C/s. After keeping the deformation temperature for 1 min, let the sample be heated evenly in the heating furnace. Keep the deformation temperature unchanged and compress at a constant strain rate. After finally reaching the set compression amount, the sample is immediately put into water for water quenching to retain the high-temperature tissue of the sample. The deformation temperatures are 900 °C, 1000 °C, and 1100 °C, and the strain rate are $\;0.01\;s^{-1}$, $0.1\;s^{-1}$, $1\;s^{-1}$.

### 3.2. Ultrasonic-Assisted Tensile Experiments

**Figure 3:**
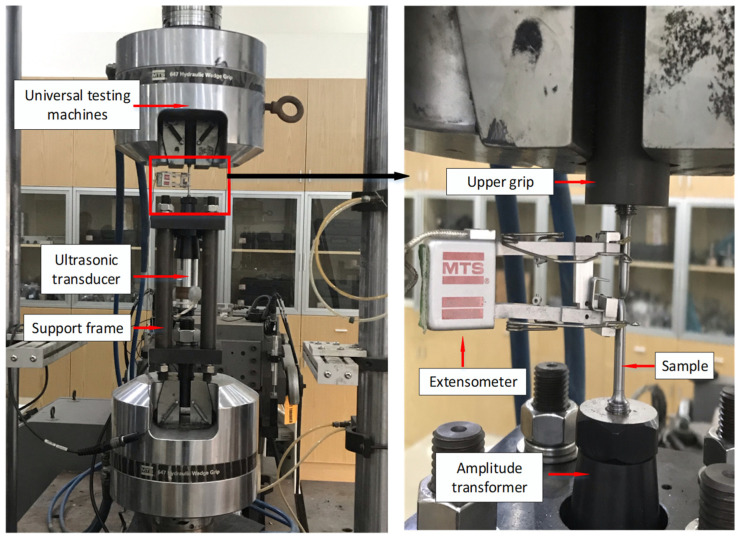
Ultrasonic-assisted tensile test device.

The ultrasonic-assisted unidirectional tensile experiment was performed on the T-table testing machine MTS 322 which is produced by MTS Systems Company Limited in the US, and the experimental device is shown in Figure 3. During the experiment, the T-type testing machine workbench clamps the lower collet and vise clamps the upper collet. The vise moves up at a certain stretching speed, and the sample is gradually stretched. At the same time, certain vibration frequencies and the amplitude of the workpiece are transferred by the transducer and amplitude rod to realize ultrasonic vibration stretching. Finally, the force and displacement curves under different stretching velocities, amplitudes, and ultrasonic durations were obtained using force sensors and displacement sensors on the test machine.

**Figure 4:**
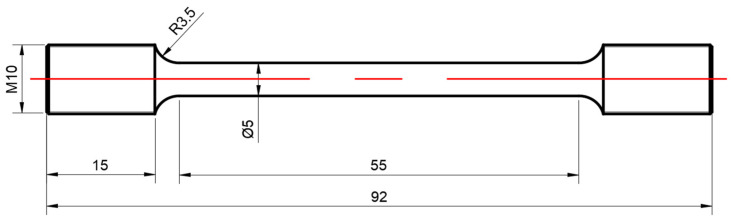
Sample.

The tensile samples were designed based on the configuration of the ultrasonic-assisted tensile device and the design standard GB/T 2281.1—2010. The tensile specimen is a rod-length specimen with a diameter of 5 mm and a threaded connection at both ends, as shown in Figure 4. The ultrasonic vibration frequency of the ultrasonic-assisted unidirectional tensile experiment is 20 kHz, and the experimental scheme is shown in the Table 2:

## 4. Results and Discussion

### 4.1. Hot Compression Experimental Results

**Figure 5:**
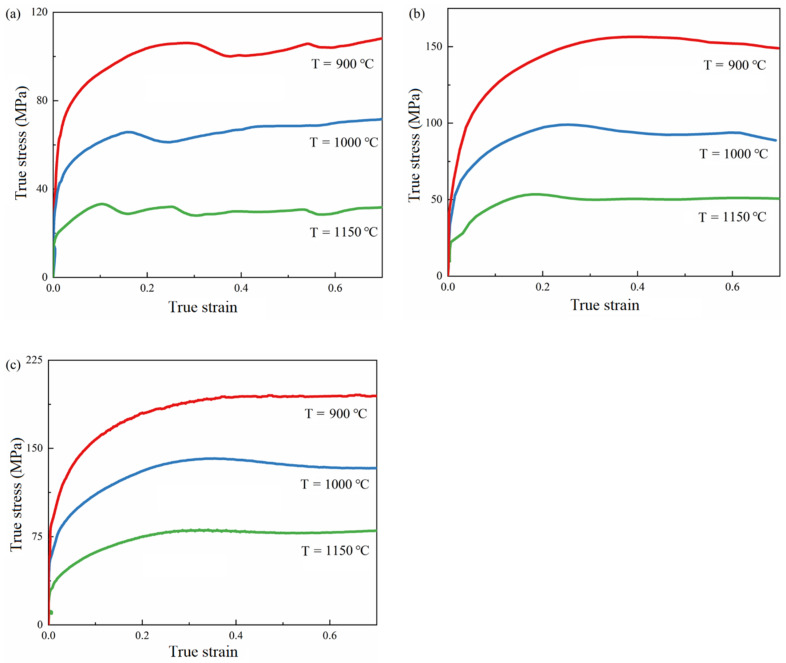
Flow stress curve of 9310 steel. (**a**) $\dot{\varepsilon }=0.01\;s^{-1}$; (**b**) $\dot{\varepsilon }=0.1\;s^{-1}$; (**c**) $\dot{\varepsilon }=1{\;s}^{-1}$.

The flow stress curves at different deformation temperatures and strain rates are obtained by hot compression, as shown in Figure 5.

It can be seen that the deformation temperature and strain rate have an obvious influence on the flow stress of 9310 steel. With an increase in deformation temperature and a decrease in strain rate, the flow stress decreases. The true stress–strain curve has the characteristics of peak stress, strain softening, and steady flow.

At large strain rates, the flow stress curve first increases and then tends to be stable without single or double peaks. When the strain is small, the flow stress curve increases because of the effect of work hardening. During this period, dislocation multiplication, dislocation density, and flow stress increase. Then, the flow stress increases and slows down because of the softening effect. When the deformation energy storage caused by dislocation multiplication increases to a certain extent, dislocation cells and subgrain boundaries are formed, and softening is mainly based on dynamic recovery. Finally, the steady state is caused by the counterbalance between work hardening and softening and the balance between the multiplication and recovery of dislocation.

When the strain rate decreases, the flow stress curve first increases and then decreases to a steady state with the characteristic of a “single peak.” When the strain rate continued to decrease, the true stress–strain curve appeared to be a “multi-peak” phenomenon. The “single peak” characteristic of the flow stress curve is that material softening dominates after the flow stress reaches its maximum value, and the material is significantly softened. At this time, softening has two behaviors: dynamic recovery and recrystallization. Under high temperature and low strain rates, softening is dominant, dynamic recrystallization occurs, the peak stress is significantly reduced, and the softening phenomenon is significant. Since the deformation temperature exceeds the recrystallization temperature, dislocation multiplication at the early stage of deformation promotes the occurrence of recrystallization. When the dislocation proliferates to a certain extent, dynamic recrystallization occurs, and recrystallized grains are generated. The dislocations in the material were reduced, and the flow stress curve was reduced. Finally, the rate of dislocation multiplication and annihilation reaches equilibrium, and the flow stress enters the steady-state stage. The occurrence of “multi-peak” characteristics means that there is continuous dynamic recrystallization in the process.

### 4.2. Ultrasonic-Assisted Tensile Experiment Results

**Figure 6:**
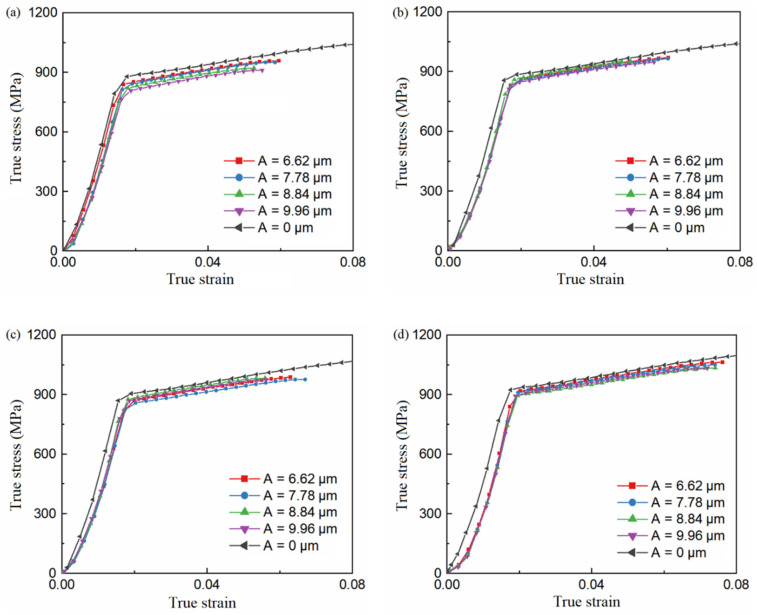
True stress–strain curve (**a**) $\dot{\varepsilon }=0.0003\;s^{-1}$ (**b**) $\dot{\varepsilon }=0.003{\;s}^{-1}$ (**c**) $\dot{\varepsilon }=0.03\;s^{-1}$ (d) $\dot{\varepsilon }=0.3\;s^{-1}$.

The data obtained from the results of the ultrasonic-assisted tensile experiment are shown in Figure 6.

The yield strength, tensile strength, and fracture strength were extracted from the experimental results, and the amplitude of the stress decrease caused by ultrasound was calculated using Equation (28). λ is the average value of the ratio of stress decrease caused by ultrasound at the stage of uniform plastic deformation.

(28) $$\lambda =\frac{\Delta \sigma }{\sigma_{C}}=\frac{\sigma_{C}-\sigma_{U}}{\sigma_{C}}$$

where $\sigma_{C}$ is the flow stress under ordinary tension, and $\sigma_{U}$ is the flow stress under ultrasonic-assisted tension.

**Figure 7:**
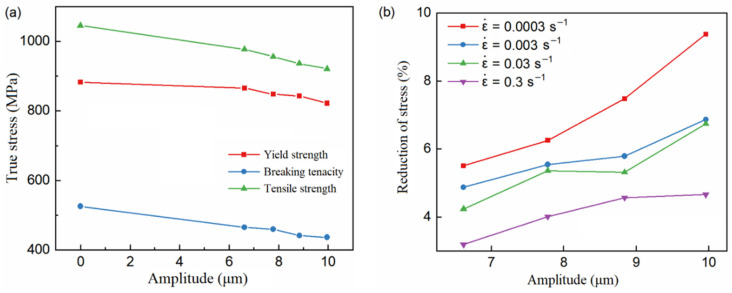
The variation curve of the stress and the stress reduction amplitude with the amplitude. (**a**) Stresses at different amplitudes. (**b**) Stress reduction amplitude at different amplitudes.

Figure 7 shows the yield strength, fracture strength, tensile strength, and stress decrease amplitude under different ultrasonic amplitudes and without ultrasonic when the strain rate is 0.0003. It can be seen that ultrasonic treatment effectively reduces yield strength, fracture strength, and tensile strength. With an increase in amplitude, the stress decreases, and the amplitude of stress decreases with an increase in ultrasonic amplitude, and the relationship is linear.

**Figure 8:**
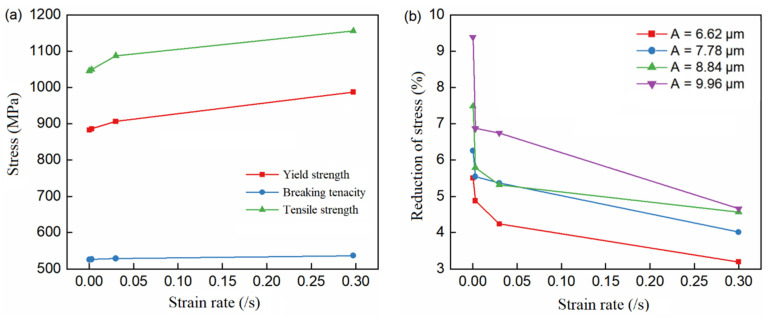
Variation curve of stress values and stress reduction amplitude with strain rate. (**a**) Stresses at different strain rates. (**b**) Stress reduction amplitude at different strain rates.

Figure 8 shows the variation curve of true stress and stress reduction amplitude with strain rate. It can be seen that all stress values increase with the increase in strain rate. At room temperature, the softening effect of ultrasound decreases with an increase in the strain rate. Thermal activation at room temperature is not sufficient to cause dislocation migration, and work hardening at high strain rates is more significant than that at low strain rates. Therefore, the flow stress at a high strain rate is higher than that at a low strain rate. At high strain rates, ultrasonic vibration can promote more dislocation migration, and higher work hardening can increase flow stress and reduce the ultrasonic softening effect. With the increase in strain rate, the softening effect coefficient of ultrasonic showed a nonlinear decreasing trend. 

### 4.3. Stress Reduction Amplitude

**Figure 9:**
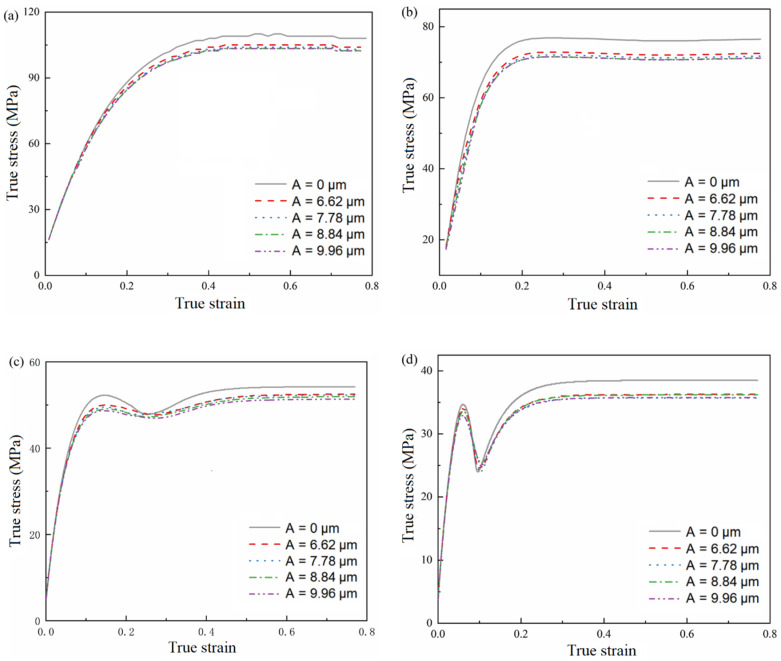
Simulated stress–strain curves at 1000 °C (**a**) $\dot{\varepsilon }=0.3{\;s}^{-1}$ (**b**) $\dot{\varepsilon }=0.03{\;s}^{-1}$ (**c**) $\dot{\varepsilon }=0.003{\;s}^{-1}$ (**d**) $\dot{\varepsilon }=0.0003{\;s}^{-1}$.

**Figure 10:**
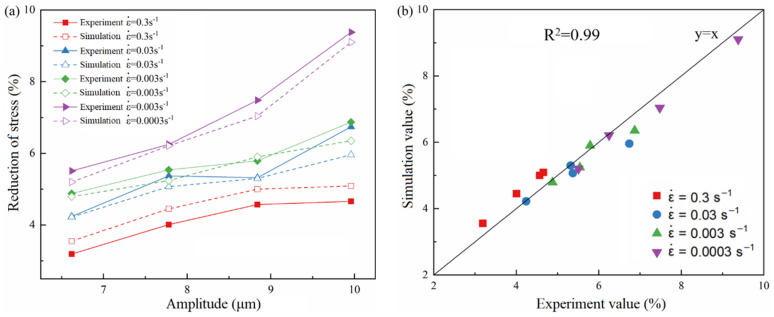
Comparison between simulated and experimental values of stress reduction amplitude. (**a**) Experimental and simulated values for stress reduction amplitude. (**b**) Linear correlation between experimental values and simulated values.

Combined with the theoretical model of ultrasonic softening and the CA model of the metal hot-forming process, the microstructure of ultrasonic-assisted hot forming was simulated. The true stress–strain curves obtained by the CA simulation of ultrasound-assisted thermoforming under different strain rates are shown in Figure 9. The comparison of stress reduction amplitude between the CA simulation and ultrasonic tensile experiment is shown in Figure 10, and the relative errors are shown in Table 3. It can be seen from the figure that the stress reduction amplitude of the ultrasonic-assisted thermal forming simulation and ultrasonic tensile experiment is close. There is a strong linear relationship between them, and the linear correlation coefficient (R^2^) is 0.99. The maximum error obtained is 11.60%, and the overall average error is 5.66%. This indicates that stress reduction occurs in the process of compression and tension under the action of ultrasound, and it is found that the amplitude of stress reduction is similar under the same ultrasonic amplitude. It can be seen that when the strain rate is high, the differences between the simulation results and the experimental results are larger than the small strain rate. This is because when the strain rate is high, the strain variable in one time step of the CA simulation will increase, which will lead to the decrease of the total simulation steps, and the decrease of the simulation accuracy.

### 4.4. CA Simulation Results

#### 4.4.1. Grain Growth Results

**Figure 11:**
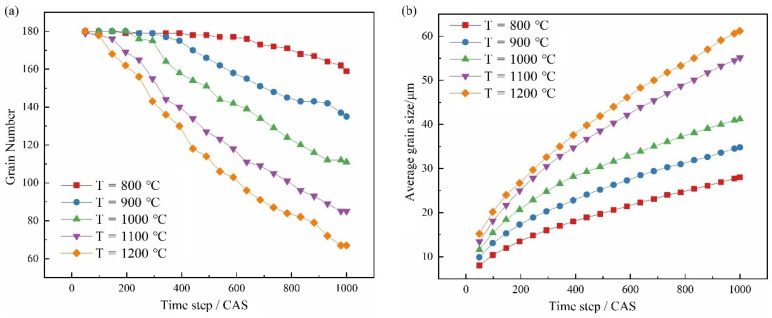
Grain growth results at different temperatures. (**a**) Variation in grain number. (**b**) Variation of average grain size.

In order to study the evolution of microstructure in the process of grain growth, the variation of grain number with time step at different temperatures is shown in Figure 11a. The relationship between average grain size and simulation step size at different temperatures is shown in Figure 11b.

It can be seen from Figure 11a that, at the same temperature, the number of grains decreases when the time step increases. At the same time, the number of grains decreases when the temperature increases. This is because grain boundary energy is the driving force of grain growth and can promote grain proliferation. When the grains grow, the grain boundary length per unit area decreases due to a decrease in grain number, which makes the total free energy in the system decrease, and the system is easier to achieve stability.

It can be seen from Figure 11b that under the same deformation temperature, the average grain size increases with an increase in time step, but the growth rate becomes slower. At the same time, the average grain size is larger with a higher deformation temperature. This is because heat can provide energy for grain boundary migration, and grains with higher deformation temperatures grow faster. However, as the grain grows, the driving force of grain boundary migration becomes smaller and smaller, and the growth rate gradually flattens.

#### 4.4.2. Effect of Initial Grain Size

**Figure 12:**
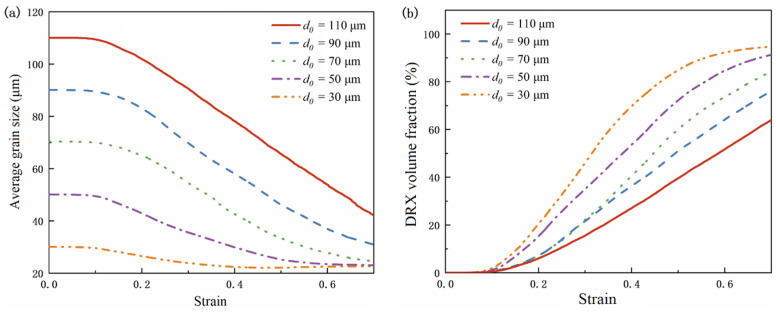
Effect of initial grain size. (**a**) Effect on average grain size. (**b**) Effect on recrystallization volume fraction.

**Figure 13:**
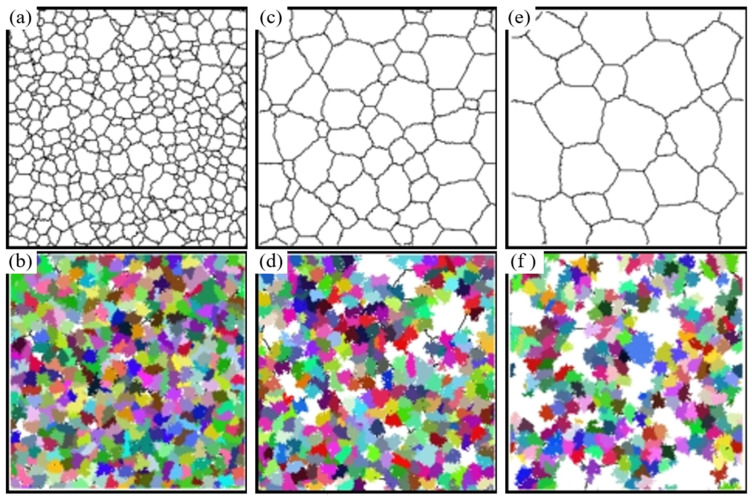
Simulation of microstructure evolution at different initial grain sizes. (**a**) Initial microstructure at initial grain size of 30 μm. (**b**) Recrystallized microstructure at initial grain size of 30 μm. (**c**) Initial microstructure at initial grain size of 70 μm. (**d**) Recrystallized microstructure at initial grain size of 70 μm. (**e**) Initial microstructure at initial grain size of 110 μm. (**f**) Recrystallized microstructure at initial grain size of 110 μm.

The initial grain size of dynamic recrystallization was set as 30 μm, 50 μm, 70 μm, 90 μm and 110 μm, deformation temperature is 1000 °C, the strain rate is 0.1 s^−1^, and the curve of average grain size and recrystallization volume fraction with the original grain size is shown in Figure 12. The microstructure image obtained by simulation is shown in Figure 13. The black part represents the grain boundary, the white part represents the initial structure, and the color part represents the grain with dynamic recrystallization.

It can be seen that with the decrease in the initial grain size, faster dynamic recrystallization occurs, and the faster it reaches stability, the volume fraction of dynamic recrystallization increases, the average grain size decreases, and the more grains have dynamic recrystallization. With the increase of strain, the average grain size first stays constant, then decreases to stable, and the volume fraction of dynamic recrystallization remained constant at first, then increased to stable. This indicates that no dynamic recrystallization occurs when deformation is small. With an increase in deformation, the dislocation density increases. When the dislocation density increases to a certain degree, the dynamic recrystallized grains are first nucleated at the grain boundaries, with a higher dislocation density. Thus, dislocation density and stress are reduced. When the dynamic recrystallized grain grows to a certain extent and the dislocation density reaches saturation, it no longer grows. The manifestation is stress stabilization, which is the softening effect of dynamic recrystallization behavior.

#### 4.4.3. Effects of Temperature and Strain Rate

**Figure 14:**
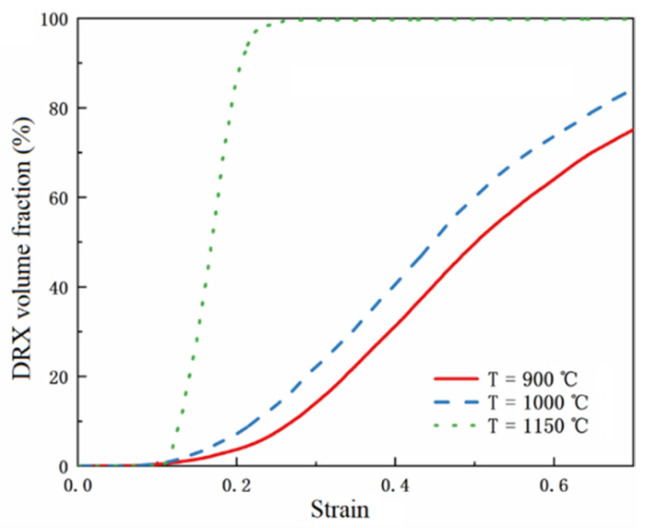
Simulated curve of the dynamic recrystallization volume fraction at different temperatures.

Figure 14 shows the simulated strain curve of the dynamic recrystallization volume fraction at different temperatures when the strain rate is 0.1 s^−1^. With the increase in strain, the volume fraction of dynamic recrystallization first remained constant and then increased to stable. It can be seen that dynamic recrystallization easily occurs at high temperatures. Under the same strain, the volume fraction of dynamic recrystallization increases with an increase in temperature. The higher the temperature, the earlier the dynamic recrystallization time, and the shorter the stable time. This is because the growth rate of dislocations is temperature dependent. The increase in temperature leads to the rapid growth of dislocation, the short incubation time of new cells, and the rapid formation of recrystallized grains, so the volume fraction of dynamic recrystallization increases.

**Figure 15:**
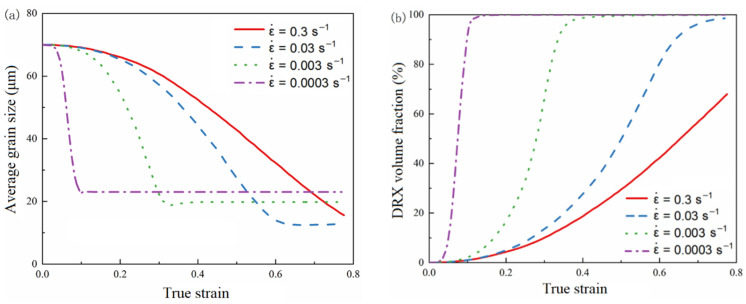
The simulation results at a temperature of 1000 °C and a vibration amplitude of 7.78 μm. (**a**) The curve of the average grain size with the true strain. (**b**) The curve of the recrystallization volume fraction with the true strain.

**Figure 16:**
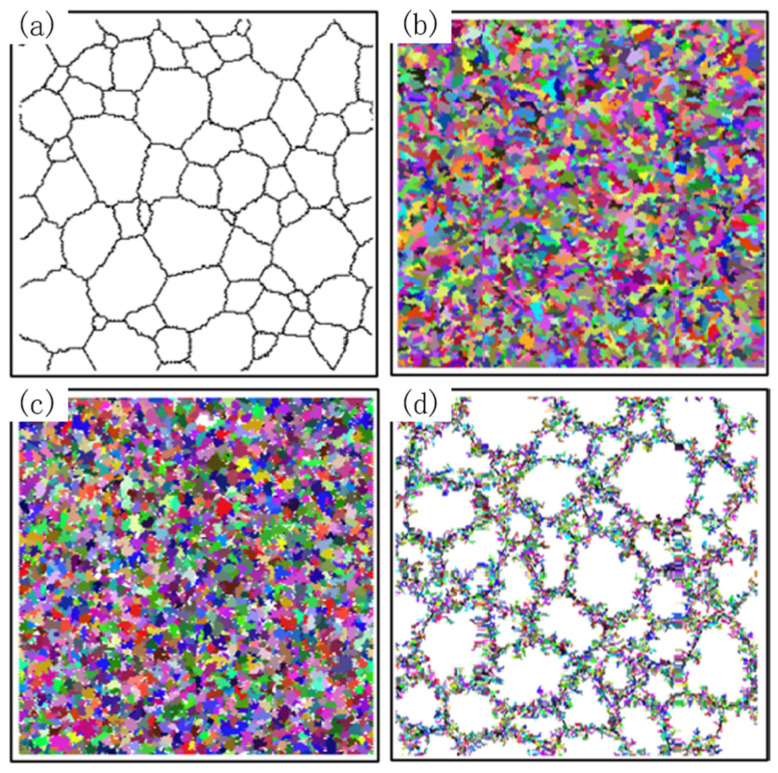
Initial microstructure and simulated microstructure results at different strain rates. (**a**) Initial microstructure. (**b**) $\dot{\varepsilon }=0.003{\;s}^{-1}$ (**c**) $\dot{\varepsilon }=0.03{\;s}^{-1}$ (**d**) $\dot{\varepsilon }=0.3{\;s}^{-1}$.

At a temperature of 1000 °C and a vibration amplitude of 7.78 μm, the variation curves of the average grain size and recrystallization volume fraction obtained by simulation with the true strain are shown in Figure 15, and the microstructure images obtained by simulation are shown in Figure 16.

It can be seen that when the strain rate decreases, the average grain size decreases to stable faster, the final recrystallized grain size is larger, the recrystallized volume fraction increases faster, and the number of recrystallized grains in the final structure is larger.

At a strain rate of 0.3 s^−1^, the recrystallized grains show a chain-like development. This is because the nucleation rate is dependent on the strain rate. When the strain rate is high, the nucleation probability also increases, and more recrystallization is generated, which makes it easy to contact each other and stop growth. When the strain rate is large, the grain does not have enough time to grow, and the dynamic recrystallization is not sufficient, so the final average grain size is large, and the recrystallization volume fraction is small.

**Figure 17:**
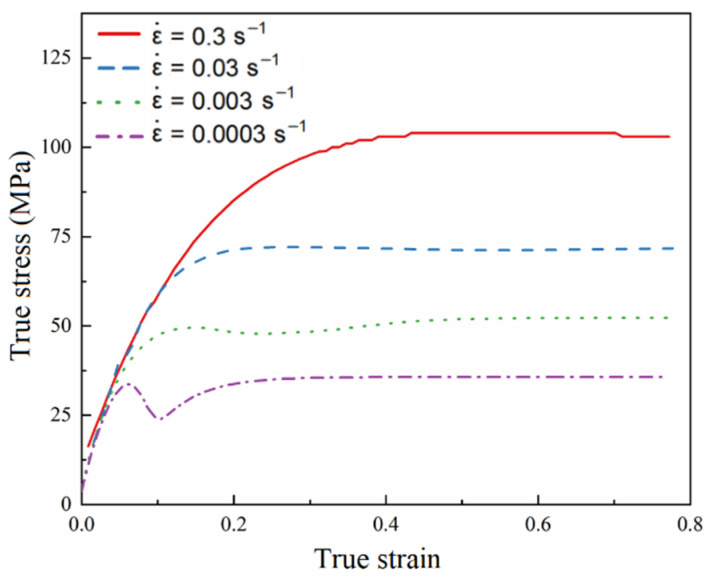
Simulated stress–strain curve at a temperature of 1000 °C and a vibration amplitude of 7.78 μm.

At a temperature of 1000 °C and a vibration amplitude of 7.78 μm, the simulated true stress–strain curves under different strain rates are shown in Figure 17. It can be seen that true stress decreases with a decrease in the strain rate. In addition, when the strain rates are 0.3 s^−1^ and 0.03 s^−1^, the true stress–strain curves show the characteristics of dynamic recovery, which indicates that material softening mainly depends on dynamic recovery. When the strain rate is small, the true stress–strain curve presents a “single peak” curve characteristic. When the strain rate decreases, the “peak” appears earlier, which indicates that dynamic recrystallization behavior is more likely to occur when the strain rate is small, which is consistent with the test results.

#### 4.4.4. Effect of Ultrasonic Vibration Amplitude

**Figure 18:**
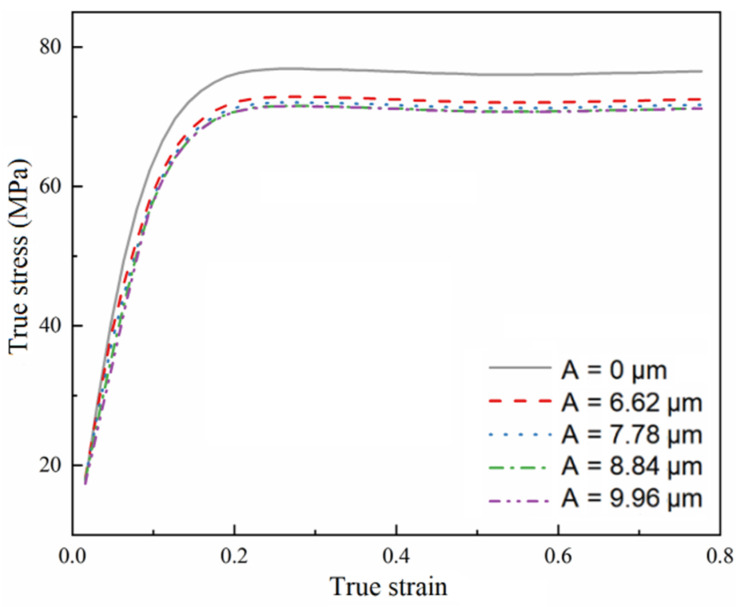
The stress–strain curve simulated at a strain rate of 0.03 s^−1^ and a temperature of 1000 °C.

At a strain rate of 0.003 s^−1^ and a temperature of 1000 °C, the simulated true stress–strain curve with vibration amplitude is shown in Figure 18. It can be seen that under different vibration amplitudes, the true stress decreases with the increase in vibration amplitude, the material is softened to different degrees, and the ultrasonic softening degree increases with the increase in vibration amplitude.

**Figure 19:**
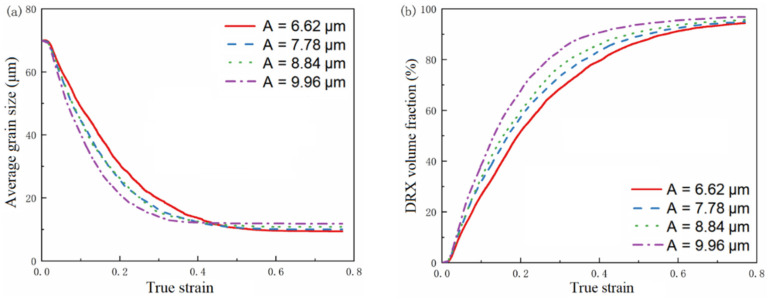
The simulation results at a strain rate of 0.03 s^−1^ and a temperature of 1000 °C. (**a**) The curve of the average grain size with the true strain. (**b**) The curve of the recrystallization volume fraction with the true strain.

**Figure 20:**
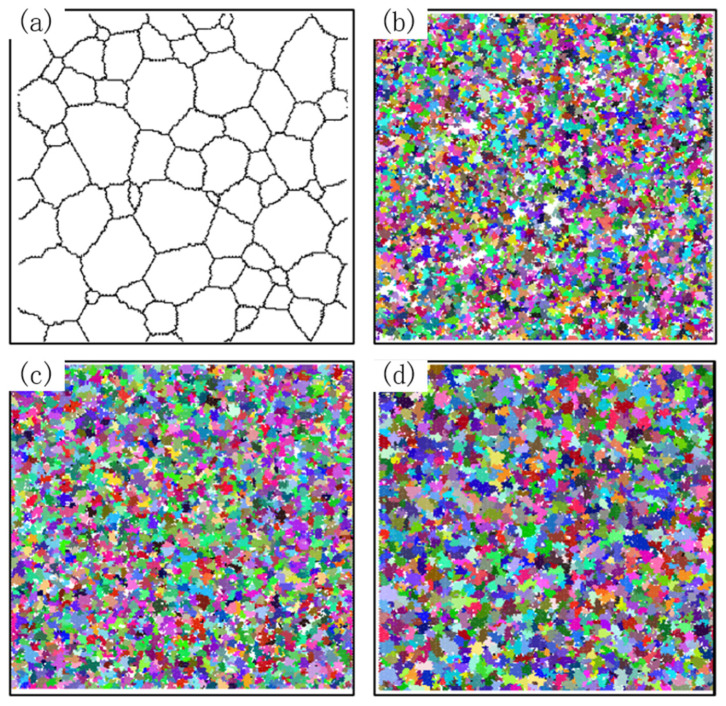
Initial microstructure and simulated microstructure results at different amplitudes. (**a**) Initial microstructure. (**b**) Simulated microstructure result when A = 0. (**c**) Simulated microstructure result when A = 6.62 μm. (**d**) Simulated microstructure result when A = 9.96 μm.

At a strain rate of 0.003 s^−1^ and a temperature of 1000 °C, the variation curves of average grain size and recrystallization volume fraction with vibration amplitude obtained by simulation are shown in Figure 19, and the microstructure image is shown in Figure 20.

Figure 19a shows that at the early stage of dynamic recrystallization, when the ultrasonic vibration amplitude increases, the average grain size decreases faster, and the average grain size curve reaches stability faster. In the late stage of dynamic recrystallization, the average grain size increases with an increase in vibration amplitude. It can be seen from Figure 19b that with the increase in ultrasonic vibration amplitude, the earlier dynamic recrystallization occurs, the more grains under dynamic recrystallization. Figure 20 shows that the number of recrystallized grains and the degree of recrystallization increase after ultrasonic vibration is applied. When the vibration amplitude is 9.96 μm, the dynamic recrystallization behavior occurs most thoroughly, and the recrystallization volume fraction reaches 99.6%.

This indicates that the introduction of ultrasonic energy reduces the activation energy of the dislocation motion. The increase of vibration amplitude and the increase of ultrasonic energy promote the occurrence of recrystallization, which leads to the faster decrease of average grain size and the faster increase of recrystallization volume fraction so that the grain is refined. Therefore, when the vibration amplitude is large, dynamic recrystallization occurs in advance, the recrystallization time is longer, the grain can grow fully, and the average grain size also increases.

## 5. Conclusions

In this paper, ultrasonic-assisted thermal forming technology is taken as the research object, based on hot compression experiments, ultrasonic tensile experiments, and cellular automaton simulations. The simulation model of ultrasonic auxiliary hot pier thickness is established based on ultrasonic-assisted thermal forming technology, and the reasons for the reduction of flow stress are explained from the microscopic scale. The main conclusions are as follows:

(1) Based on the dislocation density model, grain growth model, and dynamic recrystallization rule, ultrasonic energy was introduced to modify the thermal activation energy and dynamic recovery coefficient, and the CA model of microstructure simulation of the ultrasonic-assisted thermal forming process was established.
(2) Stress reduction occurs in the process of compression and tension under the action of ultrasound, and the stress reduction amplitude is similar under the same ultrasonic amplitude.
(3) The stress curves at different temperatures and strain rates were obtained by hot compression experiments, and it was found that the true stress–strain curves had the characteristics of peak stress, strain softening, and steady flow. At high temperatures and low strain rates, the softening phenomenon is more significant.
(4) After applying ultrasonic vibration, the introduction of ultrasonic energy reduces the activation energy of dislocation movement, promotes the occurrence of dynamic recrystallization, and finally leads to the reduction of flow stress and material softening. The softening effect is enhanced with an increase in ultrasonic vibration amplitude.
(5) Decreasing the strain rate and increasing the temperature or vibration amplitude will lead to a faster increase in the recrystallization volume fraction, and an increase in the dynamic recrystallization volume fraction and the average grain size.

## Figures and Tables

**Table 1:** Chemical composition of steel in 9310 (mass fraction, %).

C	Si	Mn	S	P	Cr	Ni	Mo	Cu
0.08	0.20	0.58	<0.015	0.0026	1.31	3.33	0.10	0.015

**Table 2:** Ultrasonic-assisted tensile test scheme.

Ultrasound Amplitude/μm	6.62	7.78	8.84	9.96
Strain rate/s^−1^	0.0003	0.003	0.03	0.3

**Table 3:** Error analysis of stress reduction amplitude.

Strain Rate (s^−1^).	Ultrasound Amplitude (μm).	Experimental Value (%)	Simulated Value (%)	Relative Error (%)
0.0003	6.62	5.51	5.20	5.57
0.0003	7.78	6.25	6.21	0.73
0.0003	8.84	7.48	7.04	5.97
0.0003	9.96	9.38	9.10	2.99
0.003	6.62	4.88	4.79	1.88
0.003	7.78	5.54	5.24	5.33
0.003	8.84	5.79	5.90	1.79
0.003	9.96	6.87	6.35	7.53
0.03	6.62	4.24	4.22	0.48
0.03	7.78	5.37	5.07	5.53
0.03	8.84	5.32	5.30	0.34
0.03	9.96	6.74	5.96	11.60
0.3	6.62	3.19	3.55	11.07
0.3	7.78	4.01	4.45	11.04
0.3	8.84	4.57	5.00	9.50
0.3	9.96	4.66	5.09	9.22

## Data Availability

The data presented in this study are available on request from the corresponding author.

## Nomenclature

**Symbol**	**Description**	**Symbol**	**Description**
$L_{ca}$	Cell size	$\theta_{m}$	Orientation angle of large Angle grains
A	Material parameter	$\theta_{i}$	Orientation difference
T	Temperature	b	Berkovian vector
Qb	Thermal diffusion activation energy	$Q_{b}^{\prime }$	Activation energy after applying ultrasonic vibration
R	Molar gas constant		
$E_{i}$	Grain boundary energy of austenite	$C_{2}$	Correction coefficient of ultrasonic energy
J	Grain boundary energy coefficient	$E_{u}$ *A*	Ultrasonic energy Ultrasonic vibration amplitude
*N*	Number of neighbors of the cellular		
*K*	Neighbor of the current judged cell	ω	Vibration circle frequency
δ	Kronecher symbol	f	Ultrasonic vibration frequency
$C_{i}$	Current orientation of the cell	$\rho_{1}$	Density
$C_{k}$	Orientation value of the neighbor cell of the current judgment cell	$M_{u}$	Grain boundary migration energy under ultrasonic vibration
ρ	Dislocation density	$k_{2u}$	Dislocation extinction coefficient under ultrasonic vibration
$k_{1}$	Dislocation increment coefficient		
$k_{2}$	Dislocation extinction coefficient	ζ	Correlation coefficient of dislocation extinction coefficient under ultrasonic vibration
*C*	Hardening index		
σ	Flow stress	η $\rho_{s}$	Exponent of the ultrasonic vibration amplitude Saturation dislocation density
α	Material constant of flow stress		
μ	Shear modulus	$P_{N}$ $\dot{N_{l}}$	Nucleation probability Grain boundary nucleation rate
$\bar{\rho }$	Average dislocation density		
$\rho_{0}$	Initial dislocation density	L	Growth distance
$\sigma_{0}$	Initial yield stress	V	Growth rate
Δt	Time step	P	Driving force
$\dot{\varepsilon }$	Strain rate	$\rho_{m}$	Dislocation density of adjacent cells
$\gamma_{i}$	Grain boundary energy of recrystallization	$\rho_{rex}$	Dislocation density of recrystallized grains respectively
M	Grain boundary migration energy		
τ	Dislocation line energy	$r_{i}$	Radius of the recrystallization grain
$D_{ob}$	Self-diffusion coefficient	$L_{max}$	Maximum growth distance
$\gamma_{m}$	Grain boundary energy of large angle grains		
